# Identification of Potential Antimicrobial Targets of Pseudomonas aeruginosa Biofilms through a Novel Screening Approach

**DOI:** 10.1128/spectrum.03099-22

**Published:** 2023-02-13

**Authors:** Jules D. P. Valentin, Stefanie Altenried, Adithi R. Varadarajan, Christian H. Ahrens, Frank Schreiber, Jeremy S. Webb, Henny C. van der Mei, Qun Ren

**Affiliations:** a Laboratory for Biointerfaces, Empa, Swiss Federal Laboratories for Materials Science and Technology, St. Gallen, Switzerland; b University of Groningen and University Medical Center Groningen, Department of BioMedical Engineering, Groningen, Netherlands; c Molecular Ecology, Agroscope and Swiss Institute of Bioinformatics, Zurich, Switzerland; d Division of Biodeterioration and Reference Organisms (4.1), Department of Materials and the Environment, Federal Institute for Materials Research and Testing (BAM), Berlin, Germany; e Institute for Life Sciences, University of Southampton, Southampton, United Kingdom; f National Biofilms Innovation Centre, University of Southampton, Southampton, United Kingdom; University at Albany

**Keywords:** biofilm formation, biofilm tolerance, PA0720, FliC, PA3785

## Abstract

Pseudomonas aeruginosa is an opportunistic pathogen of considerable medical importance, owing to its pronounced antibiotic tolerance and association with cystic fibrosis and other life-threatening diseases. The aim of this study was to highlight the genes responsible for P. aeruginosa biofilm tolerance to antibiotics and thereby identify potential new targets for the development of drugs against biofilm-related infections. By developing a novel screening approach and utilizing a public P. aeruginosa transposon insertion library, several biofilm-relevant genes were identified. The Pf phage gene (*PA0720*) and flagellin gene (*fliC*) conferred biofilm-specific tolerance to gentamicin. Compared with the reference biofilms, the biofilms formed by *PA0720* and *fliC* mutants were completely eliminated with a 4-fold-lower gentamicin concentration. Furthermore, the *mreC*, *pprB*, *coxC*, and *PA3785* genes were demonstrated to play major roles in enhancing biofilm tolerance to gentamicin. The analysis of biofilm-relevant genes performed in this study provides important novel insights into the understanding of P. aeruginosa antibiotic tolerance, which will facilitate the detection of antibiotic resistance and the development of antibiofilm strategies against P. aeruginosa.

**IMPORTANCE** Pseudomonas aeruginosa is an opportunistic pathogen of high medical importance and is one of the main pathogens responsible for the mortality of patients with cystic fibrosis. In addition to inherited antibiotic resistance, P. aeruginosa can form biofilms, defined as communities of microorganisms embedded in a self-produced matrix of extracellular polymeric substances adhering to each other and/or to a surface. Biofilms protect bacteria from antibiotic treatments and represent a major reason for antibiotic failure in the treatment of chronic infections caused by cystic fibrosis. Therefore, it is crucial to develop new therapeutic strategies aimed at specifically eradicating biofilms. The aim of this study was to generalize a novel screening method for biofilm research and to identify the possible genes involved in P. aeruginosa biofilm tolerance to antibiotics, both of which could improve the understanding of biofilm-related infections and allow for the identification of relevant therapeutic targets for drug development.

## OBSERVATION

Since the 1950s, antimicrobial drugs have been mainly developed in screens using planktonic bacteria (1). However, more than 80% of human chronic infections are associated with biofilms (2). Biofilms are communities of microorganisms embedded in a self-produced matrix of extracellular polymeric substances adhering to each other and/or to a surface (2). Sessile bacteria in biofilms are protected from immune system defenses and can tolerate up to 1,000 times higher antibiotic concentrations than planktonic cells, requiring doses that cannot be administered in humans (3). New therapeutic options are urgently needed to combat biofilm-related infections, especially those caused by the multidrug-resistant pathogen Pseudomonas aeruginosa (4, 5). We hypothesized that studying biofilm-relevant genes would advance the mechanistic understanding of P. aeruginosa biofilm tolerance to antibiotics and highlight new targets for antibiofilm drug development. This approach differed from other global screenings by rationally reducing the number of analyzed genes, allowing a more extensive characterization of their roles in biofilms.

**FIG 1:**
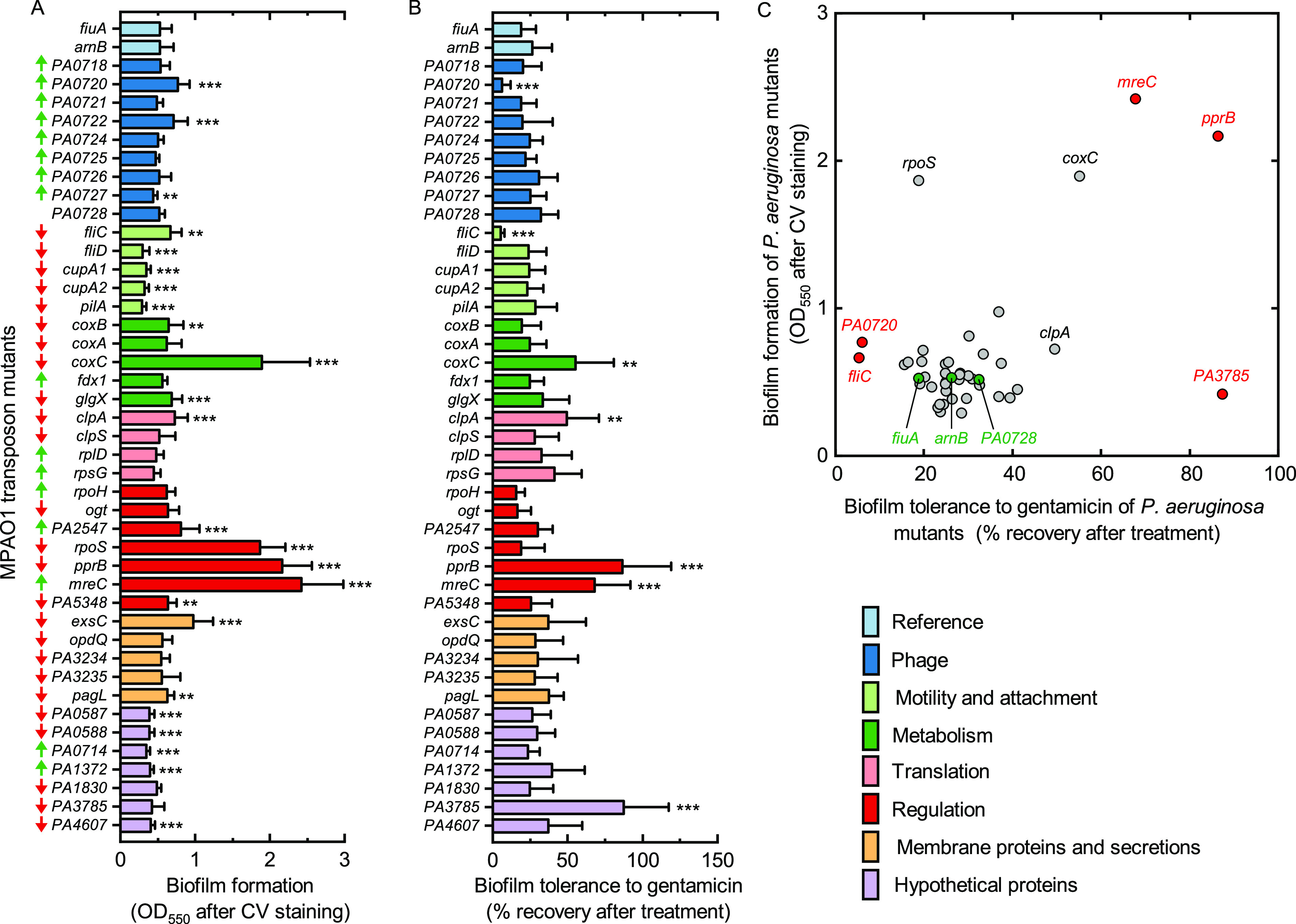
Influence of biofilm-associated genes on biofilm formation (A) and biofilm tolerance to gentamicin (B) in P. aeruginosa MPAO1. (A) Biofilm biomass was quantified using crystal violet (CV) staining after 24 h of growth in M9 medium under static conditions at 37°C. (B) Biofilm tolerance to gentamicin was quantified by measuring the turbidity of the biofilm suspension after 24 h of gentamicin treatment at 100 μg/mL and 24 h recovery in fresh M9 medium. Biofilm recovery was expressed relative to untreated biofilms (defined as 100%). The results represent the means ± standard deviations (SD) of two independent biological repeats (three for the *fiuA*, *arnB*, and *PA0720* mutants), with eight (A) and four (B) technical repeats each. Student *t* tests were performed using the biomass and recovery of the *fiuA* mutant as references. **, *P < *0.01; ***, *P < *0.001. The arrows in front of each gene indicate whether the gene is upregulated (green) or downregulated (red) in P. aeruginosa biofilm cells compared with planktonic cells (6). (C) The phenotypic distribution of all tested P. aeruginosa mutants (obtained by combining the results from panels A and B). The green symbols represent the mutants used as references. The red symbols indicate mutants with significantly different biofilm tolerance to gentamicin, compared with the reference *fiuA* mutant (*P < *0.001).

Biofilm-relevant genes were selected from a transcriptomic study by Whiteley et al., in which 73 genes were upregulated or downregulated by more than 2-fold in P. aeruginosa biofilms compared with planktonic cells (6). Of the 73 genes, 42 were functionally characterized using a P. aeruginosa MPAO1 transposon mutant library (7) and our *in vitro* assay system (8, 9) (see Table S1 in the supplemental material for additional information on the selected transposon mutants). The antibiotic tolerance of P. aeruginosa biofilms was tested using gentamicin, an aminoglycoside commonly used to treat P. aeruginosa infections (10) (Fig. 1 and Table 1), and the last-resort antibiotic colistin (Fig. S1; see also Fig. S2 to S4 for detailed results). To account for the potential influence of the transposon Tn*5* background (9), MPAO1 mutants missing the *fiuA* and *arnB* genes, which encode a receptor for heterologous siderophores (11) and colistin resistance (12), respectively, were selected as control reference strains. As previously shown (9, 13), the inactivation of *fiuA* or *arnB* in P. aeruginosa MPAO1 did not impact biofilm formation and tolerance toward gentamicin and led to phenotypes representative of most analyzed mutants (Fig. 1).

**TABLE 1:** Gentamicin susceptibility of the biofilm and planktonic cells of P. aeruginosa MPAO1 transposon mutants missing a functional *fiuA*, *PA0720*, *fliC*, *coxC*, *pprB*, or *PA3785* gene^*a*^

Gene inactivated in P. aeruginosa MPAO1	MBC-P of gentamicin (μg/mL)	MBC-B of gentamicin (μg/mL)
*fiuA*	4	1,600
*PA0720*	4–8	400
*fliC*	4–8	400
*coxC*	4	800
*pprB*	8	1,600
*PA3785*	ND	1,600

a The MBC of gentamicin for planktonic cells (MBC-P) was determined by spotting the cell suspension on brain heart infusion (BHI) agar after gentamicin treatment. The MBC of gentamicin for biofilm cells (MBC-B) was determined by spotting the cell suspension on BHI agar after gentamicin treatment and recovery. Results presented are means from two independent experiments with two technical repeats each. ND, not determined.

### Mutants with increased biofilm formation and tolerance to gentamicin.

The screening identified several genes that promoted biofilm formation and tolerance to gentamicin once inactivated. Mutations in the genes encoding the rod shape-determining protein MreC (14), the response regulator PprB (15), and the cytochrome *c* oxidase subunit CoxC (16) increased the P. aeruginosa biofilm biomass by approximately 4-fold compared with that of the reference mutant (Fig. 1A). These three mutants also showed high biofilm recovery after treatment with 100 μg/mL gentamicin (Fig. 1B). Further characterizations revealed similar minimal bactericidal concentrations of biofilms (MBC-B) of gentamicin for *fiuA* and *pprB* mutants (Table 1) but a significantly higher recovery of *pprB* mutants after exposure to a sub-MBC-B of gentamicin (Fig. S2 and S3). The latter observation concurred with previous studies which showed that *pprB* overexpression increased membrane permeability and aminoglycoside susceptibility (15). The *coxC* mutant exhibited a lower MBC-B of gentamicin than the *fiuA* mutant (Table 1), a similar MBC of planktonic cells (MBC-P), and higher biofilm recovery after exposure to a sub-MBC-B of gentamicin. Inactivating the genes encoding the aa_3_-type cytochrome *c* oxidase (i.e., *coxB* and *coxA*) did not influence biofilm formation or tolerance to gentamicin (Fig. 1). Extensive research is needed to decipher the precise roles of *mreC*, *pprB*, and *coxC* in antibiotic resistance and biofilm formation. However, our results suggest that the decreased *pprB* and *coxC* expression levels in P. aeruginosa biofilms (6) represent an active mechanism of tolerance against gentamicin.

### Mutants with altered tolerance to gentamicin but unchanged biofilm formation.

Our screening results revealed that the inactivation of the genes encoding the hypothetical protein PA3785, the bacteriophage protein PA0720, and the flagellin FliC significantly altered biofilm tolerance to gentamicin independently to biofilm biomass and growth rate (Fig. 1, Fig. S4, and Table 1).

The conserved hypothetical protein encoded by the *PA3785* gene appeared to be important for biofilm tolerance to gentamicin (Fig. 1B). Despite the unchanged MBC-B value, *PA3785* mutant biofilms exhibited the highest recovery among all tested mutants after exposure to a sub-MBC-B of gentamicin (Fig. 1B and Fig. S2). The *PA3785* gene was downregulated in P. aeruginosa biofilms compared with planktonic cells and upregulated 5-fold higher in tobramycin-treated biofilms than in untreated biofilms (6). Its exact function remains unknown, and its role in P. aeruginosa remains to be elucidated through further research.

Filamentous Pf1-like bacteriophages (Pf phages) play major roles in biofilm physiology and antibiotic tolerance (17, 18) and correlate with increased antibiotic resistance in P. aeruginosa isolates from patients with cystic fibrosis (CF) (19). Encoding a single-stranded DNA binding protein, *PA0720* is part of the Pf phage operon integrated in the P. aeruginosa genome (20). Our study suggested that *PA0720* confers biofilm-mediated tolerance of P. aeruginosa MPAO1 to gentamicin. Inactivating *PA0720* did not impact the planktonic resistance toward gentamicin but led to a 4-fold decrease in the MBC-B (Table 1). Gentamicin tolerance was only reduced by inactivating *PA0720* but not *PA0728*, which is essential to produce Pf phages (21), or any other Pf phage genes (Fig. 1B). Therefore, these results highlighted the potential role of *PA0720* in P. aeruginosa physiology, besides its role in Pf phage production. It is especially interesting that several transcriptomic and proteomic studies have found *PA0720* to be one of the few genes systematically upregulated in P. aeruginosa biofilms (6, 22–24). In summary, *PA0720* represents a promising target for drug development and has potential value as a clinically relevant marker for prediction of P. aeruginosa biofilm tolerance to gentamicin.

Our screening further revealed that the inactivation of *fliC*, which encodes flagellin type B, decreased the P. aeruginosa biofilm tolerance to gentamicin (Fig. 1B and Fig. S3). The MBC-B of gentamicin was 4-fold lower for the *fliC* mutant (400 μg/mL) than for the *fiuA* mutant (1,600 μg/mL), while the MBC-Ps were similar (Table 1). The biofilm tolerance to colistin was not affected by the inactivation of *fliC* (Fig. S1), which suggested a tolerance mechanism specific to gentamicin. In contrast to other motility gene mutants, *fliC* inactivation did not reduce the biofilm biomass of P. aeruginosa (Fig. 1A), in agreement with the findings of a previous study which showed that the nonmotile *fliC* mutant produced higher biofilm biomass, owing to an increased ability to adhere on abiotic surfaces compared to the wild type (25). *fliC* is downregulated in biofilms (6) and chronic CF infections (26), which has been attributed to an adaptive response to avoid phagocytic recognition and clearance (26). We hypothesize that *fliC* repression contributes to the biofilm-specific tolerance to antibiotics. However, further work is required to understand the precise role of FliC in biofilm physiology and assess its potential value for developing antibiofilm strategies.

### Mutants with altered biofilm formation but unchanged antibiotic tolerance.

The potential link between the antimicrobial resistance (AMR) phenotype and biofilm production is controversial (27, 28). Our results revealed that no clear correlation exists. Some mutants showed antibiotic tolerance relating to higher biofilm production, whereas others did not follow this trend (Fig. 1C). Concurring with a previous study (25), our screening showed that, compared with the reference mutant, the mutations in the motility genes (*fliD*, *cupA1*, *cupA2*, and *pilA*) led to significantly less biofilm biomass (Fig. 1A) but did not alter biofilm tolerance to gentamicin (Fig. 1B). Moreover, mutation of the gene encoding the sigma factor RpoS (29) increased the biofilm biomass by 250% (Fig. 1A) but did not increase tolerance to gentamicin (Fig. 1B) and displayed high sensitivity to colistin compared with the reference mutant (Fig. S1). These results suggested that biofilm biomass alone is not a good indicator for the AMR phenotype.

### Conclusion.

This study developed a novel screening method for biofilm research and identified candidate genes involved in biofilm antibiotic tolerance, thereby improving the understanding of biofilm-related infections and identifying relevant therapeutic targets. The screening results suggested that the level of biofilm biomass or planktonic cell resistance of a given strain is not a strong indicator of antibiotic failure. The inactivation of the Pf phage *PA0720* and flagellin *fliC* significantly reduced the gentamicin tolerance of P. aeruginosa biofilms, without impacting the biofilm biomass or MBC-P. This study discovered that novel factors such as *pprB*, *coxC*, and *PA3785* are involved in the gentamicin tolerance of P. aeruginosa biofilms. Thus, we have highlighted promising targets to develop antibiofilm treatments and relevant markers to predict gentamicin failure in the treatment of biofilm infections. The transposon mutant phenotypes remain to be confirmed with knockout strains. The present study highlights potential leads for future research in biofilm physiology and antibiofilm treatment.

## References

[1] Penesyan A, Gillings M, Paulsen I. 2015. Antibiotic discovery: combatting bacterial resistance in cells and in biofilm communities. Molecules 20:5286–5298. doi:10.3390/molecules20045286. 25812150 PMC6272253

[2] Costerton JW, Stewart PS, Greenberg EP. 1999. Bacterial biofilms: a common cause of persistent infections. Science 284:1318–1322. doi:10.1126/science.284.5418.1318. 10334980

[3] Lebeaux D, Ghigo J-M, Beloin C. 2014. Biofilm-related infections: bridging the gap between clinical management and fundamental aspects of recalcitrance toward antibiotics. Microbiol Mol Biol Rev 78:510–543. doi:10.1128/MMBR.00013-14. 25184564 PMC4187679

[4] Ventola CL. 2015. The antibiotic resistance crisis. Part 1: causes and threats. Pharm Ther 40:277–283. PMC4378521 25859123

[5] Moradali MF, Ghods S, Rehm BHA. 2017. *Pseudomonas aeruginosa* lifestyle: a paradigm for adaptation, survival, and persistence. Front Cell Infect Microbiol 7:39. doi:10.3389/fcimb.2017.00039. 28261568 PMC5310132

[6] Whiteley M, Bangera MG, Bumgarner RE, Parsek MR, Teitzel GM, Lory S, Greenberg EP. 2001. Gene expression in *Pseudomonas aeruginosa* biofilms. Nature 413:860–864. doi:10.1038/35101627. 11677611

[7] Jacobs MA, Alwood A, Thaipisuttikul I, Spencer D, Haugen E, Ernst S, Will O, Kaul R, Raymond C, Levy R, Chun-Rong L, Guenthner D, Bovee D, Olson MV, Manoil C. 2003. Comprehensive transposon mutant library of *Pseudomonas aeruginosa*. Proc Natl Acad Sci USA 100:14339–14344. doi:10.1073/pnas.2036282100. 14617778 PMC283593

[8] Mah T-F. 2014. Establishing the minimal bactericidal concentration of an antimicrobial agent for planktonic cells (MBC-P) and biofilm cells (MBC-B). J Vis Exp 83:e50854. doi:10.3791/50854. PMC4047662 24430536

[9] Varadarajan AR, Allan RN, Valentin JDP, Castañeda Ocampo OE, Somerville V, Pietsch F, Buhmann MT, West J, Skipp PJ, van der Mei HC, Ren Q, Schreiber F, Webb JS, Ahrens CH. 2020. An integrated model system to gain mechanistic insights into biofilm-associated antimicrobial resistance in *Pseudomonas aeruginosa* MPAO1. NPJ Biofilms Microbiomes 6:46. doi:10.1038/s41522-020-00154-8. 33127897 PMC7603352

[10] Bassetti M, Vena A, Croxatto A, Righi E, Guery B. 2018. How to manage *Pseudomonas aeruginosa* infections. Drugs Context 7:212527. doi:10.7573/dic.212527. 29872449 PMC5978525

[11] Llamas MA, Sparrius M, Kloet R, Jiménez CR, Vandenbroucke-Grauls C, Bitter W. 2006. The heterologous siderophores ferrioxamine B and ferrichrome activate signaling pathways in *Pseudomonas aeruginosa*. J Bacteriol 188:1882–1891. doi:10.1128/JB.188.5.1882-1891.2006. 16484199 PMC1426570

[12] Fernández L, Gooderham WJ, Bains M, McPhee JB, Wiegand I, Hancock REW. 2010. Adaptive resistance to the “last hope” antibiotics polymyxin B and colistin in *Pseudomonas aeruginosa* is mediated by the novel two-component regulatory system ParR-ParS. Antimicrob Agents Chemother 54:3372–3382. doi:10.1128/AAC.00242-10. 20547815 PMC2916309

[13] Valentin JDP, Straub H, Pietsch F, Lemare M, Ahrens CH, Schreiber F, Webb JS, van der Mei HC, Ren Q. 2022. Role of the flagellar hook in the structural development and antibiotic tolerance of *Pseudomonas aeruginosa* biofilms. ISME J 16:1176–1186. doi:10.1038/s41396-021-01157-9. 34880458 PMC8940932

[14] Wachi M, Doi M, Okada Y, Matsuhashi M. 1989. New *mre* genes *mreC* and *mreD*, responsible for formation of the rod shape of *Escherichia coli* cells. J Bacteriol 171:6511–6516. doi:10.1128/jb.171.12.6511-6516.1989. 2687239 PMC210540

[15] Wang Y, Ha U, Zeng L, Jin S. 2003. Regulation of membrane permeability by a two-component regulatory system in *Pseudomonas aeruginosa*. Antimicrob Agents Chemother 47:95–101. doi:10.1128/AAC.47.1.95-101.2003. 12499175 PMC149007

[16] Alvarez-Ortega C, Harwood CS. 2007. Responses of *Pseudomonas aeruginosa* to low oxygen indicate that growth in the cystic fibrosis lung is by aerobic respiration. Mol Microbiol 65:582–582. doi:10.1111/j.1365-2958.2007.05847.x. PMC4157922 17581126

[17] Secor PR, Sweere JM, Michaels LA, Malkovskiy AV, Lazzareschi D, Katznelson E, Rajadas J, Birnbaum ME, Arrigoni A, Braun KR, Evanko SP, Stevens DA, Kaminsky W, Singh PK, Parks WC, Bollyky PL. 2015. Filamentous bacteriophage promote biofilm assembly and function. Cell Host Microbe 18:549–559. doi:10.1016/j.chom.2015.10.013. 26567508 PMC4653043

[18] Webb JS, Thompson LS, James S, Charlton T, Tolker-Nielsen T, Koch B, Givskov M, Kjelleberg S. 2003. Cell death in *Pseudomonas aeruginosa* biofilm development. J Bacteriol 185:4585–4592. doi:10.1128/JB.185.15.4585-4592.2003. 12867469 PMC165772

[19] Burgener EB, Sweere JM, Bach MS, Secor PR, Haddock N, Jennings LK, Marvig RL, Johansen HK, Rossi E, Cao X, Tian L, Nedelec L, Molin S, Bollyky PL, Milla CE. 2019. Filamentous bacteriophages are associated with chronic *Pseudomonas* lung infections and antibiotic resistance in cystic fibrosis. Sci Transl Med 11:eaau9748. doi:10.1126/scitranslmed.aau9748. 30996083 PMC7021451

[20] Alberts B, Frey L, Delius H. 1972. Isolation and characterization of gene 5 protein of filamentous bacterial viruses. J Mol Biol 68:139–152. doi:10.1016/0022-2836(72)90269-0. 4115107

[21] Mooij MJ, Drenkard E, Llamas MA, Vandenbroucke-Grauls CMJE, Savelkoul PHM, Ausubel FM, Bitter W. 2007. Characterization of the integrated filamentous phage Pf5 and its involvement in small-colony formation. Microbiology (Reading) 153:1790–1798. doi:10.1099/mic.0.2006/003533-0. 17526836 PMC3820363

[22] Sauer K, Camper AK, Ehrlich GD, Costerton JW, Davies DG. 2002. *Pseudomonas aeruginosa* displays multiple phenotypes during development as a biofilm. J Bacteriol 184:1140–1154. doi:10.1128/jb.184.4.1140-1154.2002. 11807075 PMC134825

[23] Hentzer M, Eberl L, Givskov M. 2005. Transcriptome analysis of *Pseudomonas aeruginosa* biofilm development: anaerobic respiration and iron limitation. Biofilms 2:37–61. doi:10.1017/S1479050505001699.

[24] Guilbaud M, Bruzaud J, Bouffartigues E, Orange N, Guillot A, Aubert-Frambourg A, Monnet V, Herry J-M, Chevalier S, Bellon-Fontaine M-N. 2017. Proteomic response of *Pseudomonas aeruginosa* PAO1 adhering to solid surfaces. Front Microbiol 8:1465. doi:10.3389/fmicb.2017.01465. 28824592 PMC5541441

[25] O'Toole GA, Kolter R. 1998. Flagellar and twitching motility are necessary for *Pseudomonas aeruginosa* biofilm development. Mol Microbiol 30:295–304. doi:10.1046/j.1365-2958.1998.01062.x. 9791175

[26] Wolfgang MC, Jyot J, Goodman AL, Ramphal R, Lory S, Tiedje JM. 2004. *Pseudomonas aeruginosa* regulates flagellin expression as part of a global response to airway fluid from cystic fibrosis patients. Proc Natl Acad Sci USA 101:6664–6668. doi:10.1073/pnas.0307553101. 15084751 PMC404102

[27] Gajdács M, Baráth Z, Kárpáti K, Szabó D, Usai D, Zanetti S, Donadu MG. 2021. No correlation between biofilm formation, virulence factors, and antibiotic resistance in *Pseudomonas aeruginosa*: results from a laboratory-based *in vitro* study. Antibiotics 10:1134. doi:10.3390/antibiotics10091134. 34572716 PMC8471826

[28] Karballaei Mirzahosseini H, Hadadi-Fishani M, Morshedi K, Khaledi A. 2020. Meta-analysis of biofilm formation, antibiotic resistance pattern, and biofilm-related genes in *Pseudomonas aeruginosa* isolated from clinical samples. Microb Drug Resist 26:815–824. doi:10.1089/mdr.2019.0274. 31976811

[29] Murakami K, Ono T, Viducic D, Kayama S, Mori M, Hirota K, Nemoto K, Miyake Y. 2005. Role for *rpoS* gene of *Pseudomonas aeruginosa* in antibiotic tolerance. FEMS Microbiol Lett 242:161–167. doi:10.1016/j.femsle.2004.11.005. 15621433

