# Supplementary material for: Identification of Potential Antimicrobial Targets of Pseudomonas aeruginosa Biofilms through a Novel Screening Approach
Source: Microbiol Spectr. 2023 Feb 13;11(2):e03099-22. doi: 10.1128/spectrum.03099-22 (PMC10100978; doi:10.1128/spectrum.03099-22)
Supplement: Supplemental file 1 — Supplemental material. Download spectrum.03099-22-s0001.pdf, PDF file, 1.0 MB [file spectrum.03099-22-s0001.pdf]

Supplementary Information

Identification of potential antimicrobial targets of *Pseudomonas aeruginosa* biofilms through a novel screening approach

Jules D. P. Valentin[1,2,§,*], Stefanie Altenried[1], Adithi R. Varadarajan[3], Christian H. Ahrens[3], Frank Schreiber[4], Jeremy S. Webb[5], Henny C. van der Mei[2], Qun Ren[1,*]

[1] Laboratory for Biointerfaces, Empa, Swiss Federal Laboratories for Materials Science and Technology, St. Gallen, Switzerland.

[2] University of Groningen and University Medical Center Groningen, Department of BioMedical Engineering, Groningen, Netherlands.

[3] Molecular Ecology, Agroscope and SIB Swiss Institute of Bioinformatics, CH-8820 Wädenswil, Switzerland.

[4] Federal Institute for Materials Research and Testing (BAM), Berlin, Germany.

[5] Institute for Life Sciences & National Biofilms Innovation Centre, University of Southampton, Southampton SO16 7PX, UK.

Corresponding authors:

* E-mail: jules.valentin68@gmail.com

* E-mail: qun.ren@empa.ch

Present address:

[§] Department of Chemistry, University of Fribourg, CH-1700 Fribourg, Switzerland

Table S1. Transposon mutants used from the PA Two-Allele Library. The position of the transposon in the *P. aeruginosa* genome was confirmed by the Manoil Lab using Sanger and Illumina sequencing.

ORF	Gene	Strain name	Genotype	Sanger confirmed	Illumina confirmed
**Reference**					
PA0470	*fiuA*	PW1861	PA0470-F05::ISlacZ/hah	+	+
PA3552	*arnB*	PW7021	PA3552-G12::ISphoA/hah	+	+
**Prophage Pf**					
PA0718		PW2301	PA0718-D09::ISphoA/hah	+	+
PA0720		PW2304	PA0720-E10::ISlacZ/hah	+	+
PA0721		PW2307	PA0721-F07::ISphoA/hah	+	+
PA0722		PW2309	PA0722-A10::ISlacZ/hah	+	+
PA0724		PW2311	PA0724-F12::ISlacZ/hah	+	+
PA0725		PW2313	PA0725-B09::ISlacZ/hah	+	+
PA0726		PW2316	PA0726-A09::ISlacZ/hah	+	+
PA0727		PW2320	PA0727-F05::ISlacZ/hah	+	+
PA0728		PW2321	PA0728-F01::ISphoA/hah	+	+
**Motility and attachment**					
PA1092	*fliC*	PW2971	PA1092-G10::ISphoA/hah		+
PA1094	*fliD*	PW2975	PA1094-B03::ISlacZ/hah	+	+
PA2128	*cupA1*	PW4658	PA2128-F12::ISphoA/hah	+	+
PA2129	*cupA2*	PW4659	PA2129-H06::ISlacZ/hah	+	+
PA4525	*pilA*	PW8621	PA4525-E01::ISlacZ/hah	+	+
**Metabolism**					
PA0105	*coxB*	PW1177	PA0105-F12::ISphoA/hah	+	+
PA0106	*coxA*	PW1179	PA0106-G07::ISlacZ/hah	+	+
PA0108	*coxC*	PW1184	PA0108-B07::ISlacZ/hah	+	+
PA0362	*fdx1*	PW1668	PA0362-E07::ISlacZ/hah	+	+
PA2160	*glgX*	PW4697	PA2160-G04::ISlacZ/hah	+	+
**Translation**					
PA2620	*clpA*	PW5389	PA2620-G12::ISlacZ/hah	+	+
PA2621	*clpS*	PW5391	PA2621-F03::ISphoA/hah	+	+
PA4262	*rplD*	PW8196	PA4262-D07::ISlacZ/hah		
PA4267	*rpsG*	PW6286	PA4267-C05::ISlacZ/hah		
**Regulation**					
PA0376	*rpoH*	PW1692	PA0376-F11::ISphoA/hah	+	+
PA0995	*ogt*	PW2796	PA0995-D12::ISlacZ/hah	+	+
PA2547		PW5273	PA2547-A08::ISlacZ/hah	+	+
PA3622	*rpoS*	PW7152	PA3622-H02::ISlacZ/hah	+	+
PA4296	*pprB*	PW8238	PA4296-G05::ISphoA/hah	+	+
PA4480	*mreC*	PW8542	PA4480-F03::ISphoA/hah		+
PA5348		PW10010	PA5348-G12::ISlacZ/hah	+	+
**Membrane proteins and secretion**					
PA1710	*exsC*	PW4025	PA1710-A01::ISphoA/hah	+	+
PA3038	*opdQ*	PW6095	PA3038-D11::ISphoA/hah	+	+
PA3234		PW6418	PA3234-F05::ISlacZ/hah		
PA3235		PW6420	PA3235-E10::ISphoA/hah	+	+
PA4661	*pagL*	PW8859	PA4661-G04::ISphoA/hah	+	+
**Hypothetical proteins**					
PA0587		PW2075	PA0587-B03::ISlacZ/hah	+	+
PA0588		PW2077	PA0588-D03::ISlacZ/hah	+	+
PA0714		PW2294	PA0714-H02::ISphoA/hah	+	+
PA1372		PW3494	PA1372-F06::ISlacZ/hah	+	+
PA1830		PW4215	PA1830-D06::ISlacZ/hah	+	+
PA3785		PW7408	PA3785-A06::ISlacZ/hah	+	+
PA4607		PW8761	PA4607-E03::ISlacZ/hah	+	+

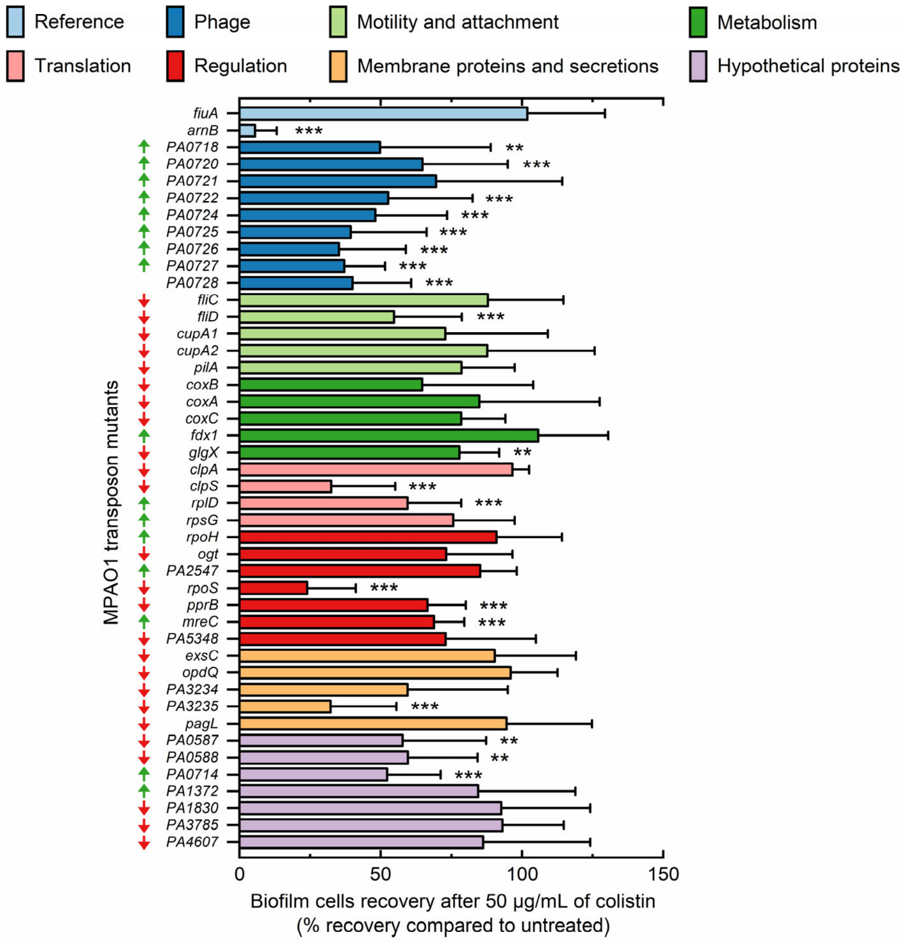

Figure S1. Influence of biofilm-associated genes on biofilm tolerance to colistin in *P. aeruginosa* MPAO1 mutant strains. Biofilm tolerance to colistin was quantified by measuring the turbidity of the biofilm suspension after 24 h colistin treatment at 50 µg/mL and 24 h recovery in fresh M9 medium. Biofilm recovery was expressed relative to untreated biofilms (defined as 100%). The results represent the mean ± standard deviation (SD) of two independent biological repeats (three for the *fiuA*, *arnB*, and *PA0720* mutants) with four technical repeats each. Student *t*-tests were performed with ** $P < 0.01$ and *** $P < 0.001$, using the recovery of the *fiuA* mutant as reference. The arrows in front of each gene indicate whether the gene is upregulated (green) or downregulated (red) in *P. aeruginosa* biofilm cells compared with planktonic cells (1).

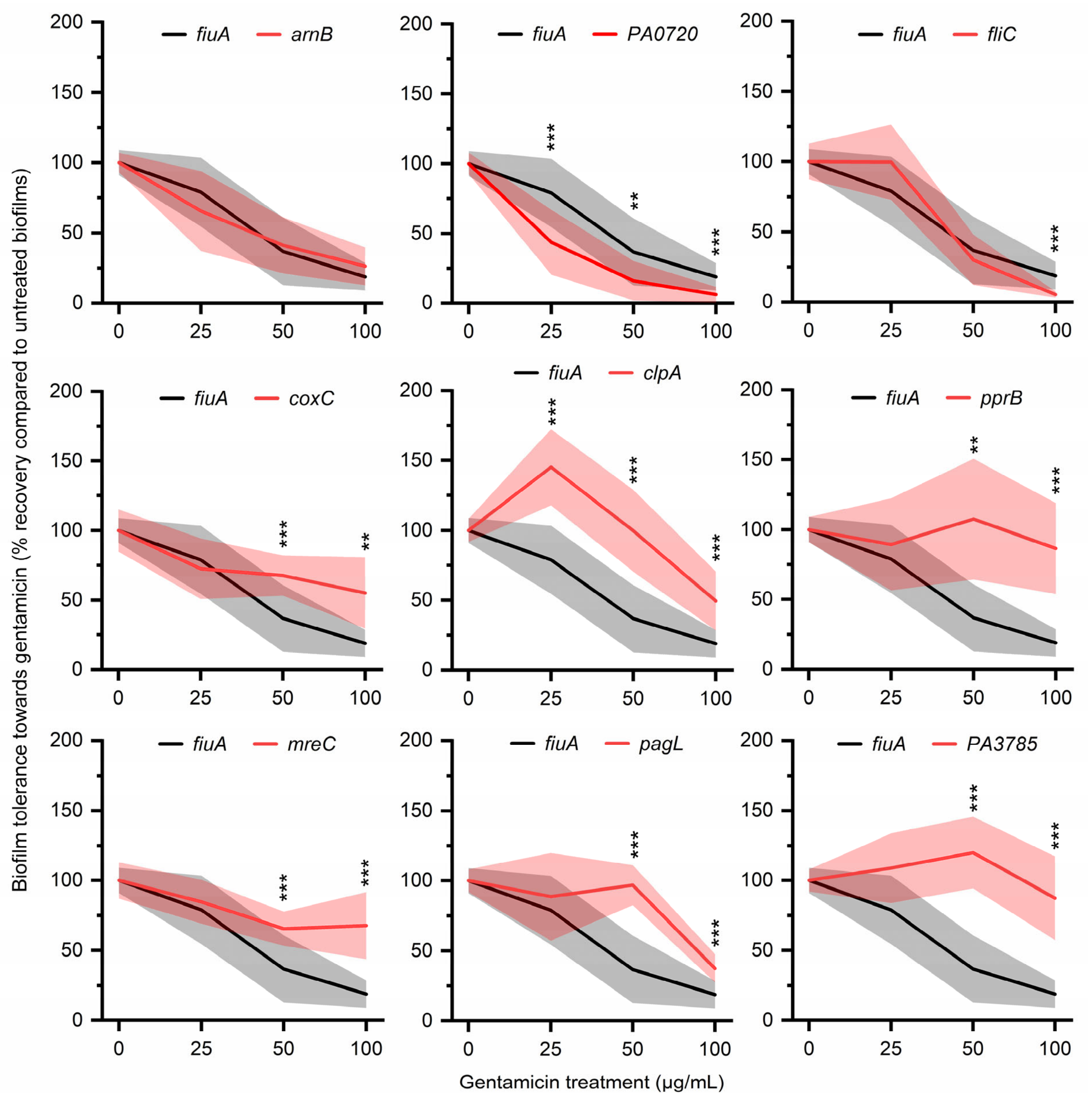

Figure S2. Influence of biofilm-associated genes on tolerance of *P. aeruginosa* MPAO1 mutants to gentamicin in the biofilm mode of growth. Biofilm tolerance to gentamicin was quantified by measuring the turbidity of the biofilm suspension after 24 h treatment with different concentrations of gentamicin and 24 h recovery in fresh M9 medium. The tolerance was expressed relative to untreated biofilms (defined as 100%). Results represent the mean ± SD of two independent biological (four for the *fiuA*, *arnB* and *PA0720* mutants) repeats with four technical repeats each, with the exception of the *pagL* and *PA3785* mutants that were assayed only once with four technical repeats at 25 and 50 µg/mL. Student *t*-tests were performed with ** $P < 0.01$ and *** $P < 0.001$ compared to the recovery of the *fiuA* mutant at each concentration.

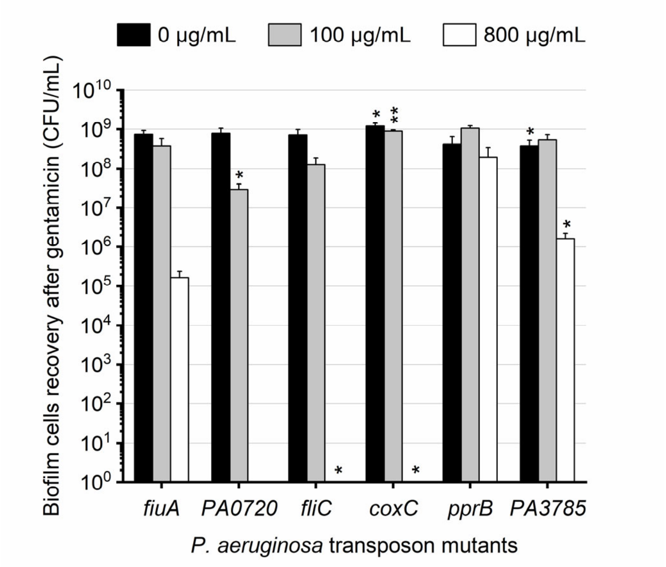

Figure S3. Gentamicin susceptibility of biofilm cells of *P. aeruginosa* MPAO1 mutants missing a functional *fiuA*, *PA0720*, *fliC*, *coxC*, *pprB* or *PA3785* gene. Biofilm tolerance to gentamicin was measured by counting the colony forming units (CFU) after 24 h treatment with gentamicin and 24 h recovery in fresh M9 medium. Results are the mean ± SD from two independent experiments with two technical repeats each. Student's *t*-tests were performed with * equals to $P < 0.05$, ** equals to $P < 0.01$ and *** equals to $P < 0.001$ compared to the recovery of the *fiuA* mutant at each gentamicin concentrations.

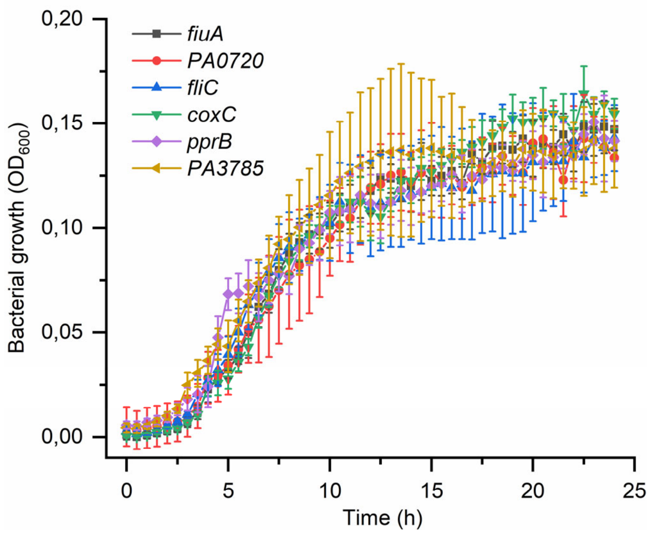

Figure S4. Growth kinetic of *P. aeruginosa* MPAO1 mutants missing a functional *fiuA*, *PA0720*, *fliC*, *coxC*, *pprB* and *PA3785* gene. Bacteria were grown in M9 medium at 37°C

under static conditions. Results represent the mean ± SD of three independent biological repeats with two technical repeats each.

Supplemental Methods

Bacterial strains and culture media. *P. aeruginosa* MPAO1 WT and transposon insertion mutants were obtained from the PA Two-Allele Library provided by the Manoil Lab (2). Bacteria were routinely grown in Brain Heart Infusion (BHI, CM1135, Oxoid, UK) broth and agar at 37°C. Biofilm and antibiotic assays were performed in 96 well plates (TPP tissue culture 96 well plates, Z707902, Sigma-Aldrich) covered with an air-permeable foil (Breathe-Easy sealing membrane, Z380059, Sigma-Aldrich) without further shaking. All assays were done in M9 medium (M9 minimal salts 5X, M6030, Sigma-Aldrich, USA) containing 48 mM Na_2HPO_4, 22 mM KH_2PO_4, 9 mM NaCl, 19 mM NH_4Cl and complemented with 2 mM $MgSO_4$ (63140-F, Sigma-Aldrich, USA) 100 µM $CaCl_2$ (21100, Fluka, Germany) and 20 mM glucose (G7528, Sigma-Aldrich, USA) (3).

Biofilm formation and tolerance to gentamicin and colistin. Biofilm tolerance of *P. aeruginosa* strains was assessed as described previously (4, 5). Overnight liquid cultures of bacteria were diluted 1:100 in M9 medium and biofilms were grown during 24 h incubation at 37°C. To quantify biofilm formation, the biofilm biomass was measured by crystal violet staining (0.1%, 30 min, 61135, Fluka, India) followed by washing with 0.9 % NaCl (71380, Sigma-Aldrich, USA), dissolution in 95 % ethanol and measurement of the optical density at 550 nm using a spectrophotometer (Genesys 6, USA). To quantify biofilm tolerance, biofilms were washed with 0.9% NaCl and incubated for 24 h at 37°C in M9 medium supplemented with increasing concentrations of gentamicin (0-100 µg/mL, G1914, Sigma-Aldrich, USA) and colistin (0-50 µg/mL, C4461, Sigma-Aldrich, China). Antibiotic solutions were then washed with 0.9 % NaCl and biofilm cells were allowed to recover in fresh M9 medium for 24 h at 37°C. The regrowth in the biofilm suspension, indicating biofilm cell tolerance, was quantified by measuring the optical density at 600 nm and by plating the suspended biofilm on agar

plates and counting the CFU per mL. Additionally, the MBC-B was measured by spotting approx. 2 μL of the biofilm suspension after antibiotic treatment and recovery on BHI agar and by determining the concentration needed to achieve complete absence of bacterial growth.

Planktonic cells resistance to gentamicin. Planktonic resistance of MPAO1 mutants was assessed by measuring the MBC-P as described previously (5). M9 media supplemented with increasing concentrations of gentamicin were inoculated with MPAO1 mutants at a final concentration of $5x10^6$ CFU/mL. After 24 h at 37°C, the MBC-P was measured by spotting approximately 2 μL of the planktonic cell suspension on BHI agar and by determining the concentration needed to achieve complete absence of bacterial growth.

Statistical analysis. All analysis were performed with Excel (Microsoft) and all data are represented as a mean ± SD. Student t-tests were performed to evaluate statistical differences between two groups (e.g. biofilm biomass of the *fiuA* and *PA0720* mutants, recovery of untreated and treated biofilm cells). All graphs were generated using Origin.

References

1. Whiteley M, Bangera MG, Bumgarner RE, Parsek MR, Teitzel GM, Lory S, Greenberg EP. 2001. Gene expression in *Pseudomonas aeruginosa* biofilms. Nature 413:860–864.

2. Jacobs MA, Alwood A, Thaipisuttikul I, Spencer D, Haugen E, Ernst S, Will O, Kaul R, Raymond C, Levy R, Chun-Rong L, Guenthner D, Bovee D, Olson MV, Manoil C. 2003. Comprehensive transposon mutant library of *Pseudomonas aeruginosa*. Proc Natl Acad Sci USA 100:14339–14344.

3. Barraud N, Hassett DJ, Hwang S-H, Rice SA, Kjelleberg S, Webb JS. 2006. Involvement of Nitric Oxide in Biofilm Dispersal of *Pseudomonas aeruginosa*. J Bacteriol 188:7344–7353.

4. Varadarajan AR, Allan RN, Valentin JDP, Castañeda Ocampo OE, Somerville V, Pietsch F, Buhmann MT, West J, Skipp PJ, van der Mei HC, Ren Q, Schreiber F, Webb JS, Ahrens CH. 2020. An integrated model system to gain mechanistic insights into biofilm-associated antimicrobial resistance in *Pseudomonas aeruginosa* MPAO1. NPJ Biofilms Microbiomes 6:46.

5. Mah T-F. 2014. Establishing the Minimal Bactericidal Concentration of an antimicrobial agent for Planktonic Cells (MBC-P) and Biofilm Cells (MBC-B). JoVE 83:e50854.
